# Propagation of dissection in a residually-stressed artery model

**DOI:** 10.1007/s10237-016-0806-1

**Published:** 2016-07-09

**Authors:** Lei Wang, Steven M. Roper, Nicholas A. Hill, Xiaoyu Luo

**Affiliations:** School of Mathematics and Statistics, University of Glasgow, Glasgow, UK

**Keywords:** Arterial dissection, Residual stress, HGO model, Soft tissue mechanics, Buckling, Tear propagation, Critical pressure, XFEM, Cohesive traction–separation law

## Abstract

This paper studies dissection propagation subject to internal pressure in a residually-stressed two-layer arterial model. The artery is assumed to be infinitely long, and the resultant plane strain problem is solved using the extended finite element method. The arterial layers are modelled using the anisotropic hyperelastic Holzapfel–Gasser–Ogden model, and the tissue damage due to tear propagation is described using a linear cohesive traction–separation law. Residual stress in the arterial wall is determined by an opening angle $\alpha$ in a stress-free configuration. An initial tear is introduced within the artery which is subject to internal pressure. Quasi-static solutions are computed to determine the critical value of the pressure, at which the dissection starts to propagate. Our model shows that the dissection tends to propagate radially outwards. Interestingly, the critical pressure is higher for both very short and very long tears. The simulations also reveal that the inner wall buckles for longer tears, which is supported by clinical CT scans. In all simulated cases, the critical pressure is found to increase with the opening angle. In other words, residual stress acts to protect the artery against tear propagation. The effect of residual stress is more prominent when a tear is of intermediate length ($\simeq$90$^\circ$ arc length). There is an intricate balance between tear length, wall buckling, fibre orientation, and residual stress that determines the tear propagation.

## Introduction

An arterial dissection is a tear within the wall of a large artery, such as the aorta. The dissection can lead to the creation of a false lumen through which blood flows, and propagation of the tear can quickly lead to death as a result of decreased blood supply to other organs, damage to the aortic valve, and rupture of the artery. The loading conditions on the arterial wall, the geometry of the artery and of the tear, and the material properties of the arterial wall determine whether the tear propagates. A prediction of how the critical condition for tear propagation depends on these factors could help to optimize diagnosis and treatment.

In the absence of loading, many biological soft tissues are not stress-free, but subject to residual stress. At physiological loading, the residual stress in arteries reduces variation in the stress distribution across the arterial wall and decreases the peak stress (Cardamone et al. 2009; Chuong and Fung 1986). Fung (1991) was the first to show that a radial cut along artery can release much of the residual stress. Hence, using an opening angle is a theoretical approach for recovering the stress-free configuration, and the value of the opening angle is often used to quantify the residual stress. For example, Holzapfel et al. (2000) employed this method to obtain the residual stress in a two-layer model of a rabbit carotid arterial wall.

**Fig. 1:**
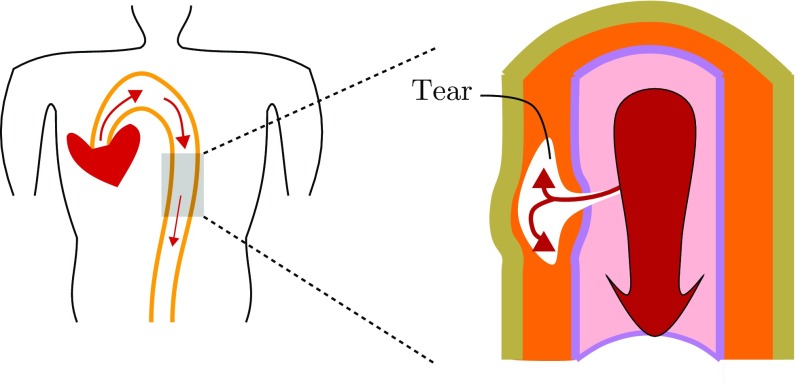
A cartoon of arterial dissection. A defect (a tear) that initially begins in the intimal layer of the artery extends into the media where it can grow to create a *false lumen*. Inflation of the false lumen by blood pressure can restrict the true lumen, restricting blood supply to major organs. In some cases, the tear can rupture the artery. The loading condition on the arterial wall, the geometry of the artery and of the tear, and the material properties of the arterial wall determine whether the tear propagates

Here we study the effects of residual stress on an arterial dissection or tear. If the artery is subject to hypertension (Golledge and Eagle 2008; Kodolitsch et al. 2000) or the arterial wall becomes weak, e.g. a defect arises in the inner surface of arterial wall, then blood at high pressure may force its way into the wall and propagate longitudinally, creating a dissection, as indicated in Fig. 1.

Tear propagation in the absence of residual stress has been studied previously. For example, Sommer et al. (2008) subjected samples of human aortic media to peeling tests to estimate dissection property. This was followed by a number of computational simulations (Ferrara and Pandolfi 2010; Gasser and Holzapfel 2006). Wang et al. (2014) and Tong et al. (2011) performed similar experiments on human coronary and human carotid arteries, respectively. In these experimental and computational studies, the tear propagation is stably driven by controlling the displacement when peeling apart strips. However, blood pressure drives the propagation of dissections in vivo (Braverman 2010; Rajagopal et al. 2007). There are fewer literatures on studying this pressure-driven propagation. In experiments on a porcine thoracic aorta subject to pressure, Carson and Roach (1990) measured the peak pressure to tear the media and the work per unit area of tissue required to propagate a tear and showed that these values are independent of the tear depth, while Tam et al. (1998) studied the effect of depth of the initial tear on the critical pressure for propagation and showed that the critical pressure decreases as the depth increases. Arterial dissection during balloon angioplasty of an atherosclerotic artery was modelled by Badel et al. (2014), in which the arterial wall is compressed by inflating a balloon controlled by a displacement boundary condition. Recently, we developed a computational scheme to compute the energy release rate, a variable for quantifying the risk of propagation, for pressure-driven dissection propagation using a nonlinear energy argument in a 2D model (Wang et al. 2015), and reported how the critical pressure for arterial dissection can be affected by fibre orientation, tear length, and surrounding tissues.

In this paper, we extend our previous work (Li 2013; Wang et al. 2015) and investigate what role the residual stress plays in the critical condition for propagation of the dissection in a two-layer (media and adventitia) arterial wall. To model the tear propagation, we use the eXtended Finite Element Method (XFEM) (Moes et al. 1999) implemented in ABAQUS (2014). For the material properties, we use the fibre-reinforced anisotropic hyperelastic Holzapfel–Gasser–Ogden (HGO) constitutive law (Holzapfel et al. 2000). We developed a computational protocol that involves a sequence of novel boundary conditions to close an opening angle, starting from the stress-free configuration, to obtain the unloaded configuration and so introduce the residual stress. Notably, the unloaded configuration is the same for different values of the opening angle, ensuring that the difference between simulations is solely due to the residual stress field. To achieve this, we solve the equilibrium equations analytically to obtain the individual stress-free configuration for different opening angles.

**Fig. 2:**
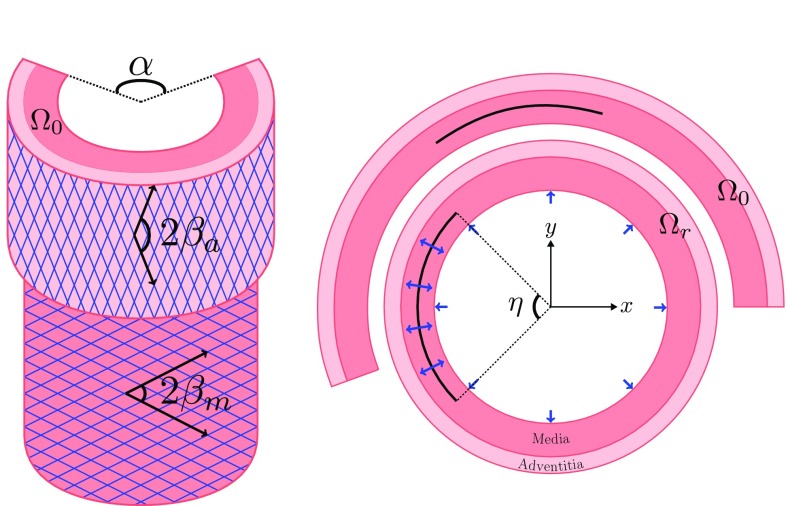
Cross sections of the arterial wall in the stress-free $\varOmega _0$ and unloaded $\varOmega _\mathrm{r}$ configurations, subject to the same pressure loading on the inner radial and the tear surfaces. *Each layer* is a hyperelastic fibre-reinforced material, modelled by the HGO strain-energy function. $\beta _\mathrm{m}$ and $\beta _\mathrm{a}$ define the angles between the two families of fibres in the media and adventitia. The tear subtends an angle $\eta$

The paper is organized as follows. In Sect. 2, we detail the arterial model including the geometry, the constitutive model, and the cohesive law. In Sect. 3, we show how to calculate analytically the stress-free configurations for different opening angles with a specified unloaded configuration, how to incorporate residual stress using ABAQUS, and how to determine the critical pressure. The results are shown in Sect. 4, followed by discussion and conclusions in Sect. 5.

## The model

### Geometry

Our model is based on the widely used two-layer rabbit carotid artery (Holzapfel et al. 2000), but includes the effects of residual stress as shown in Fig. 2. An arterial dissection is modelled as a tear in the wall of thick-walled cylinder in an unloaded and *residually-stressed* configuration $\varOmega _\mathrm{r}$. The residual stress field in $\varOmega _\mathrm{r}$ is calculated from the value of the opening angle $\alpha$ in the *zero-stress* configuration $\varOmega _0$. The configurations are listed in Table 1. The data for $\varOmega _0$ are obtained from (Holzapfel et al. 2000), and the data for $\varOmega _\mathrm{r}$ are computed using the analytical approach to be discussed below. We introduce an idealized dissection (Fig. 2) along an arc of constant radius in $\varOmega _\mathrm{r}$, which is connected to the lumen of the vessel by negligibly small tears, so that the dissection surfaces are subject to the same blood pressure as in the true lumen.

**Table 1:** The geometry of the two-layer arterial wall, $t_\mathrm{m}$ and $t_\mathrm{a}$ are the wall thickness of the media and adventitia

Configurations	$r_\mathrm{i}\,(\hbox {mm})$	$t_\mathrm{m}\,(\hbox {mm})$	$t_\mathrm{a}\,(\hbox {mm})$	$\alpha \,(^\circ )$
$\varOmega _0$	1.430	0.260	0.130	160
$\varOmega _\mathrm{r}$	0.739	0.259	0.120	0

### Constitutive law

The description for the two layers of the arterial wall is the same except for different values of parameters in each layer. For the mechanical response of the arterial wall, we use the incompressible Holzapfel–Gasser–Ogden (HGO) constitutive law (Holzapfel et al. 2000), with strain-energy function

1 $$\begin{aligned} W = W_{m}({I}_1) + W_{f}({I}_4, {I}_6)= c({I}_1-3) + \sum \limits _{n=4,6} w({I}_n,k_1,k_2), \end{aligned}$$

where *c*, $k_1$, $k_2$ are material parameters for each layer of the artery, as listed in Table 2 (Holzapfel et al. 2000), and $I_1, I_4$, and $I_6$ are the invariants of the right Cauchy–Green strain tensor $\mathbf {C}$ and the fibre-structure tensor $\mathbf {M}_n$, defined as

$$\begin{aligned} {I}_1 = \text {tr}\, \mathbf {{C}}, \quad {I}_n = {\mathbf {C}} :\mathbf {M}_n, \quad n=4,6, \end{aligned}$$

where $\mathbf {M}_4=\mathbf {A}_{1} \otimes \varvec{A}_{1}$ and $\mathbf {M}_6=\varvec{A}_{2} \otimes \varvec{A}_{2}$ characterize the fibre orientations, $\varvec{A}_1=\left( 0,\cos \beta , \sin \beta \right)$ and $\varvec{A}_2=\left( 0,\cos \beta , -\sin \beta \right)$, as shown in Fig. 2. The two fibre families are aligned along the two directions, $\varvec{A}_{1}$ and $\varvec{A}_{2}$, and only contribute to stress when stretched, i.e.

2 $$\begin{aligned}&w\left( I_n,k_1,k_2\right) \nonumber \\&\quad = {\left\{ \begin{array}{ll} \frac{k_1}{2k_2}\left\{ \exp \left[ k_2(I_n-1)^2 \right] -1 \right\} &{} \hbox {when } I_n>1\\ 0 &{} \hbox {when } I_n \leqslant 1. \end{array}\right. } \end{aligned}$$

We assume that the artery is infinitely long (plane-strain problem), and the *z*-component in these fibre directions is ignored. Therefore, changing $\beta$ only changes the contribution of fibres in the circumferential direction.

**Table 2:** The material parameters for the HGO strain- energy function (Holzapfel et al. 2000) and the cohesive law, for a rabbit carotid artery

	$c\,(\hbox {kPa})$	$k_1\,(\hbox {kPa})$	$k_2$	$\beta \,(^\circ )$	$T_\mathrm{c}\,(\hbox {kPa})$	$G_\mathrm{c}\,(\hbox {N/m}^2)$	$\Delta u_\mathrm{c}\,(\hbox {mm})$
Media	1.5	2.3632	0.8393	29	3	0.001	0.667
Adventitia	0.15	0.5620	0.7112	62	0.3	0.0001	0.667

### Cohesive law

The initialization and propagation of a tear is modelled using the XFEM in ABAQUS (2014). The existing tear is implemented as an initial condition. The discontinuity of the displacement field at the tear surface is modelled by adding an enrichment term $H(r)\Delta u/2$ onto the otherwise continuous displacement field, where $H(r)=\pm 1$ when $r \gtrless r_\mathrm{t}$, $r_\mathrm{t}$ is the radius of the tear surface in the configuration $\varOmega _\mathrm{r}$, and $\Delta u$ is the displacement jump.

**Fig. 3:**
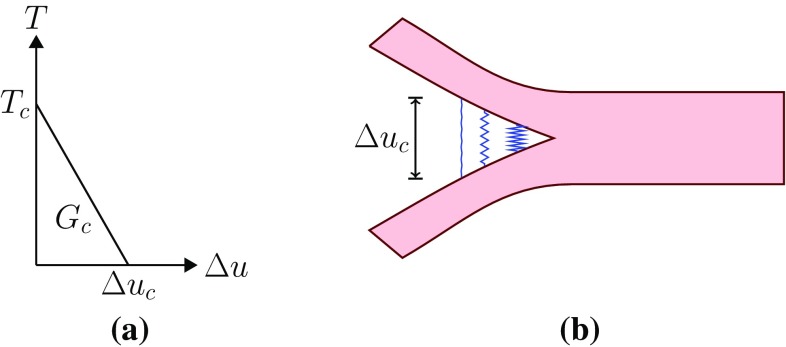
A linear cohesive law governs propagation of the dissection. **a** Cohesive law, **b** cohesive zone

We assume that the propagation of tear is governed by a linear cohesive traction–separation law (Ferrara and Pandolfi 2010) (Fig. 3), which is specified by the maximum traction $T_\mathrm{c}$ just before damage, the separation energy $G_\mathrm{c}$, and the maximum displacement jump $\Delta u_{\mathrm{c}}$, as listed in Table 2. Only two are independent. After each incremental loading step, the maximum principal stress $\sigma _{\mathrm{mp}}$ at the centroid of each element is compared to $T_\mathrm{c}$: if $\sigma _{\mathrm{mp}} \ge T_\mathrm{c}$, then the displacement jump $\Delta u$ is calculated. When $\varDelta u > \Delta u_\mathrm{c}$, the tear propagates in the direction perpendicular to the maximum tensile principal stress.

The actual value of $T_\mathrm{c}$ is material dependent and should be determined by experiments. In the absence of such data, we assume that $T_\mathrm{c}/c=2$ (where *c* is the value of the media) in this study. This, together with the assumption of the plane-strain problem, means that our computed results will be qualitative. However, different values of $T_\mathrm{c}/c$ are used later to check that the trend we observe is the same.

## Methodology

All simulations are based on a residually-stressed configuration $\varOmega _\mathrm{r}$. The residual stress and $\varOmega _\mathrm{r}$ can be obtained both analytically and numerically.

### Analytical approach

$\varOmega _\mathrm{r}$ can be obtained by setting $p_\text {i}=0$ and $\lambda _z=1$, where $p_\text {i}$ is the pressure and $\lambda _z$ is the axial stretch. Let the stress-free configuration of the artery $\varOmega _0$ be characterized in polar coordinates $(R,\varTheta )$ by

3 $$\begin{aligned} R_\text {i} \leqslant R \leqslant R_\text {o}, \qquad 0 \leqslant \varTheta \leqslant (2\pi -\alpha ), \end{aligned}$$

where $R_\mathrm{i}$, $R_\mathrm{o}$, and $\alpha$ denote the inner and outer radii, and opening angle, respectively. The corresponding unloaded configuration $\varOmega _\mathrm{r}$ in polar coordinates $(r,\theta )$ is

4 $$\begin{aligned} r_\text {i} \leqslant r \leqslant r_\text {o}, \qquad 0 \leqslant \theta \leqslant 2\pi , \end{aligned}$$

where $r_\text {i}$ and $r_\text {o}$ are the inner and outer radii of $\varOmega _\mathrm{r}$. Incompressibility requires that

5 $$\begin{aligned} r=\sqrt{\frac{R^2-R_\text {i}^2}{k}+r_\text {i}^2}, \quad \theta =k\varTheta , \end{aligned}$$

where $k=2\pi /(2\pi -\alpha )$. The deformation gradient from $\varOmega _0$ to $\varOmega _\mathrm{r}$ is

6 $$\begin{aligned} \mathbf F =\lambda _\mathrm{r} \mathbf e _\mathrm{r}\otimes \mathbf E _{R} +\lambda _\theta \mathbf e _{\theta }\otimes \mathbf E _{\varTheta } + \mathbf e _z \otimes \mathbf E _{Z}, \end{aligned}$$

where

7 $$\begin{aligned} \lambda _\mathrm{r}(R) = \frac{\partial r}{\partial R} = \frac{R}{rk}, \quad \lambda _\theta (R)=\frac{r}{R}\frac{\partial \theta }{\partial \varTheta }=\frac{kr}{R}, \end{aligned}$$

are the principal stretches. The Cauchy stress is given by

8 $$\begin{aligned} \varvec{\sigma } = -\mathfrak {p}\mathbf {I}+2\mathbf {F}\frac{\partial W}{\partial \mathbf {C}}\mathbf {F}^T, \end{aligned}$$

where $\mathfrak {p}$ is the Lagrangian multiplier associated with the incompressibility condition.

Let $\sigma _{{rr}}$ and $\sigma _{\theta \theta }$ be the radial and circumferential components of the Cauchy (residual) stress tensor in $\varOmega _\mathrm{r}$, which satisfy the momentum balance equation

9 $$\begin{aligned} \frac{\text {d}\sigma _{{rr}} }{\text {d}r} = \frac{\sigma _{\theta \theta }-\sigma _{{rr}}}{r}. \end{aligned}$$

Integration of (9) leads to

10 $$\begin{aligned} \qquad \sigma _{{rr}}(r) - \sigma _{{rr}}(r_\text {i})= \int _{\mathrm{r}_\mathrm{i}}^\mathrm{r} \left( \sigma _{\theta \theta }-\sigma _{{rr}} \right) \frac{\text {d}\tilde{r}}{\tilde{r}}, \quad r_\text {i} \leqslant r \leqslant r_\text {o}, \end{aligned}$$

Using the traction-free boundary condition

11 $$\begin{aligned} \sigma _{{rr}}(r_\text {i})=\sigma _{{rr}}(r_\text {o})=0, \end{aligned}$$

in (10), we have

12 $$\begin{aligned} \int _{\mathrm{r}_\text {i}}^{\mathrm{r}_\text {o}} \left( \sigma _{\theta \theta }-\sigma _{{rr}} \right) \frac{\text {d}r}{r} = 0. \end{aligned}$$

Substituting (5) and (8) into (12), we obtain a nonlinear integral equation, which is solved for $r_\mathrm{i}$ using Newton iteration. Substituting (1) into (8) gives

13 $$\begin{aligned} \varvec{\sigma } = -\mathfrak {p}\mathbf {I} + 2c{\mathbf {B}} + \sum \limits _{n=4,6}2w'(I_n)\mathbf {m}_n, \end{aligned}$$

where ${\mathbf {B}}=\mathbf {FF}^T$ is the left Cauchy–Green tensor, $\mathbf {m}_n=\mathbf {FM}_n\mathbf {F}^T$ is the structure tensor in the residually-stressed unloaded configuration, and $\mathfrak {p}$ is determined from (10) and (11)

14 $$\begin{aligned} \mathfrak {p}(r) = -\sigma _{{rr}}(r)+2c\lambda _\mathrm{r}^2(r). \end{aligned}$$

Equations (8) and (14) determine the Cauchy stress components $\sigma _{\theta \theta }$ and $\sigma _{zz}$.

To ensure that the difference between various simulations is only due to the residual stress, we can also determine $\varOmega _0$ from (5), (7), and (12), given $\alpha$ and $\varOmega _\mathrm{r}$.

### Numerical approach

**Fig. 4:**
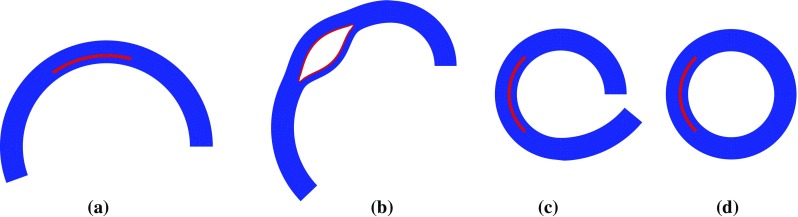
The closing process from $\varOmega _0$ to $\varOmega _\mathrm{r}$, for opening angle $\alpha =160^\circ$ and tear length $\eta =90^\circ$: **a** the stress-free configuration $\varOmega _0$, **b**, **c** move one end and pressurize both the tear and the inner radial surface, **d** residually-stressed $\varOmega _\mathrm{r}$ after the artery is closed and pressure removed

We start from the stress-free configuration $\varOmega _0$ with a specified opening angle. The numerical approach is to close the opening angle numerically and obtain the unloaded configuration $\varOmega _\mathrm{r}$. This is achieved in several steps as shown in Fig. 4. During the closing process, the inner radial and tear surface are pressurized to avoid contact. Once the ring is closed, the artificial pressure is removed. The closed configuration is then inflated to simulate the deformation of the arterial wall and dissection propagation subject to the pressure.

**Fig. 5:**
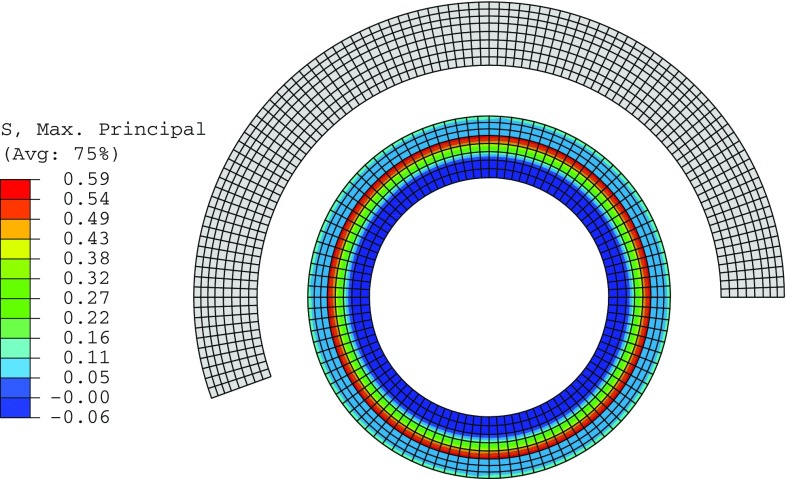
The stress-free $\varOmega _0$ (*grey*) and the unloaded $\varOmega _\mathrm{r}$ (*coloured*) configurations, where the *colour* indicates the magnitude of the maximum principal stress, which is along the circumferential direction, and the opening angle in $\varOmega _0$ is $160^\circ$

**Table 3:** Meshes used for the grid independence tests

Mesh	Nodes	Elements				Relative error in stress via Eq. (15) (%)
		Media	Adventitia	Circumference	Total	
Coarse	909	5	3	100	800	21.37
Intermediate	3417	11	5	200	3200	5.59
Fine	13,233	23	9	400	12,800	5.08

### Finite element implementation

**Fig. 6:**
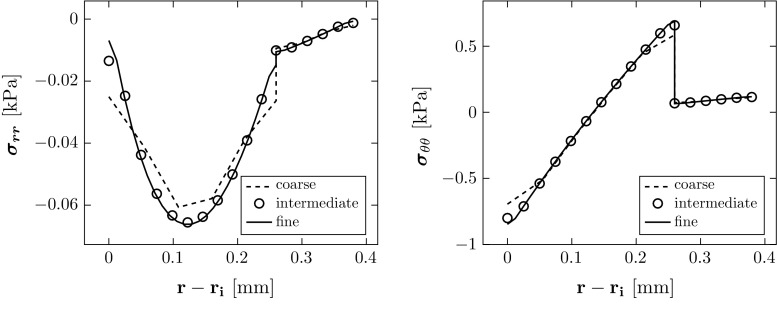
Comparison of the residual stress computed in the unloaded configuration $\varOmega _\mathrm{r}$ when $\alpha =160^\circ$. The results from the intermediate and fine meshes also overlap with the analytical solution

The computations are performed using the finite element package ABAQUS (6.13). Four-node plane-strain hybrid elements are used to construct the mesh, as shown in Fig. 5. A grid independence test was used to select the optimal number of elements (Table 3), and the computed stresses converge to the analytical solutions as shown in Fig. 6. The relative error in computed residual stress is

15 $$\begin{aligned} \text {max} \left\{ \frac{\Vert \varvec{\sigma }_{{rr}}-\varvec{\sigma }^a_{{rr}} \Vert }{\Vert \varvec{\sigma }^a_{{rr}}\Vert },\quad \frac{\Vert \varvec{\sigma }_{\theta \theta }-\varvec{\sigma }^a_{\theta \theta } \Vert }{\Vert \varvec{\sigma }^a_{\theta \theta }\Vert } \right\} , \end{aligned}$$

where $\varvec{\sigma }^a_{{rr}}$ and $\varvec{\sigma }^a_{\theta \theta }$ are the vectors of all the nodal values of the exact analytical expression for the two components of the residual stress across the wall, and $\Vert \cdot \Vert$ denotes standard $L^2$-norm, i.e. $\Vert \varvec{x}\Vert =\left( \sum _\mathrm{i} x_\mathrm{i}^2\right) ^{1/2}$ for a vector $\varvec{x}$. The intermediate mesh was then used in all the simulations for different opening angles.

## Results

### The residual stress and critical pressure

**Fig. 7:**
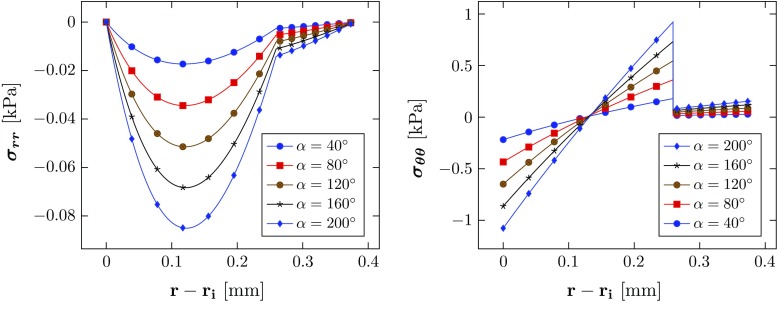
The magnitudes of residual stress components increase with the opening angle

The geometries (i.e. the radius $$R_i$$, thicknesses of the media and adventitia, $$T_m$$, $$T_a$$) of stress-free configurations $$\varOmega _0$$ associated with the *same* unloaded configuration $$\varOmega _\mathrm{r}$$ (where we specify $$\alpha $$, thicknesses of the media and adventitia, $$t_\mathrm{m}$$, $$t_\mathrm{a}$$, and inner radius $$r_\mathrm{i}$$ which are equal to $$T_m$$, $$T_a$$, and $$R_i$$ when $$a = 0)$$ are shown in Table 4. The residual stress components computed analytically are shown in Fig. 7 as a function of the opening angle $$\alpha $$. The absolute value of $$\sigma _{{rr}}$$ is greatest at the mid-radius of the media and increases with $$\alpha $$. The circumferential stress $$\sigma _{\theta \theta }$$ is in compression at the inner radius of the media and is in tension at the outer radius of the media and adventitia, and $$|\sigma _{\theta \theta }|$$ increases with $$\alpha $$. The residual stress is smaller in the adventitia.

**Fig. 8:**
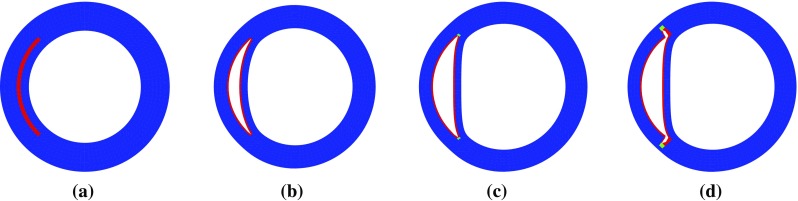
Tear propagation in an artery inflated by increased pressure loading at $\varOmega _r$ when $\alpha =160^\circ$ and $\eta =90^\circ$ for **a** the unloaded configuration $\varOmega _\mathrm{r}$ with residual stress, **b** both true and false lumens are inflated at $p/c=0.23$, **c** the tear starts to propagate at $p_\mathrm{c}/c=0.35 (=p'_\mathrm{c})$, and **d** the tear continues to propagate towards the adventitia at $p/c=0.35$. The elements in *blue* are not damaged; those in *red* are completely torn. These in other colours indicate the cohesive zone. Increasing *p* / *c* beyond the critical pressure results in a steady solution for which the tear has propagated radially outwards

**Fig. 9:**
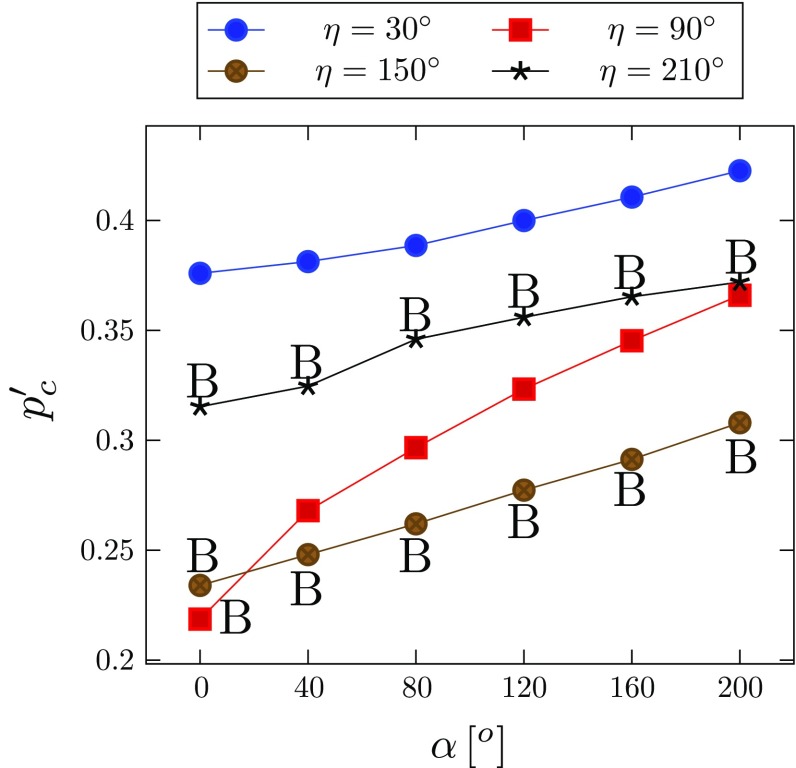
The dimensionless critical pressure $p'_\mathrm{c}$ versus the opening angle $\alpha$, for four different tear lengths $\eta$, where letter ‘*B*’ indicates that the inner wall is buckled. For discussion of different tear lengths, see Sect. 4.2

The simulated configurations for $\alpha =160^\circ$ and $\eta =90^\circ$ are shown in Fig. 8. The critical pressure at which the tear starts to propagate is identified in Fig. 8c. Since the value of $p_\mathrm{c}$ changes with the material properties, we focus on the dimensionless critical pressure $p'_\mathrm{c}=p_\mathrm{c}/c$ (where *c* is the value of the media). For the tear of length $\eta =90^\circ$, the change of $p'_{\mathrm{c}}$ with the opening angle is plotted in Fig. 9. Notice that $p'_{\mathrm{c}}$ increases with $\alpha$ in all the cases simulated, suggesting that existence of residual stress makes artery more resistant to the tear propagation.

**Table 4:** Geometries of $\varOmega _0$ (thicknesses of the media and adventitia $T_\mathrm{m}$, $T_\mathrm{a}$, and inner radius $R_\text {i}$) corresponding to the specified $\varOmega _\mathrm{r}$ and opening angle $\alpha$

$\alpha \,(^\circ )$	$R_\mathrm{i}\,(\hbox {mm})$	$T_\mathrm{m}\,(\hbox {mm})$	$T_\mathrm{a}\,(\hbox {mm})$
0	0.7395	0.2593	0.1197
40	0.8472	0.2595	0.1221
80	0.9858	0.2597	0.1246
120	1.1708	0.2599	0.1272
160	1.4300	0.2600	0.1300
200	1.8191	0.2601	0.1329

$$\varOmega _0\equiv \varOmega _r$$, when $$\alpha =0$$

### Inner wall buckling and tear length

**Fig. 10:**
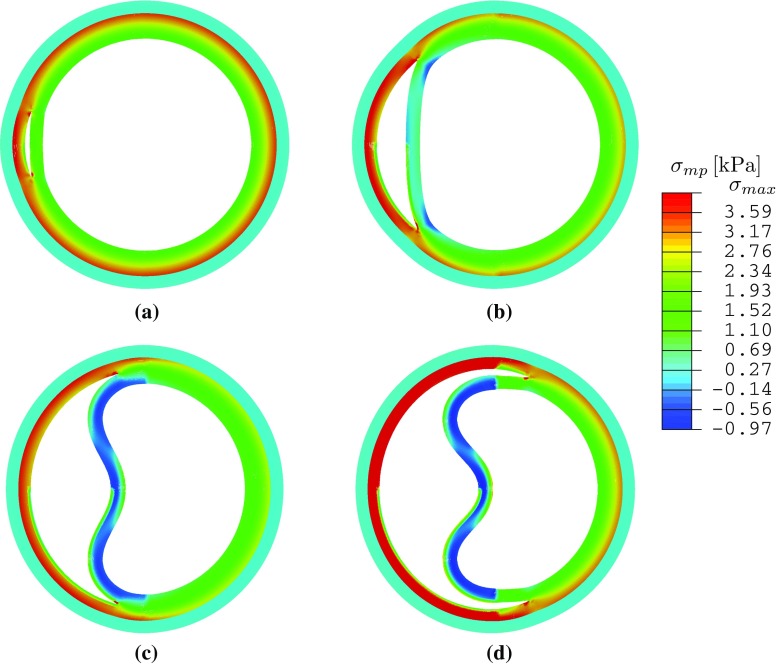
The maximum principal stress $\sigma _{\mathrm{mp}}$ is plotted in the deformed configuration for $\alpha =160^\circ$ and different lengths of tear subject to the critical pressure. Note the buckling of the inner wall for $\eta =150^\circ$ and $\eta =210^\circ$. Here $\sigma _{\mathrm{max}}$ is the averaged peak stress over the quadrature points of the adjacent elements around the tip. **a** $\eta = 30^\circ$, $p^\prime _\mathrm{c} = 0.41$, $\sigma _\mathrm{min} = -0.21$ kPa, $\sigma _\mathrm{max} = -4.18$ kPa, **b** $\eta = 90^\circ$, $p^\prime _\mathrm{c} = 0.34$, $\sigma _\mathrm{min} = -0.42$, $\sigma _\mathrm{max} = -4.86$ kPa. **c** $\eta = 150^\circ$, $p^\prime _\mathrm{c} = 0.29$, $\sigma _\mathrm{min} = -0.67$ kPa, $\sigma _\mathrm{max} = -3.96$ kPa. **d** $\eta = 210^\circ$, $p^\prime _\mathrm{c} = 0.37$, $\sigma _\mathrm{min}=-0.97$ kPa, $\sigma _\mathrm{max}= -5.09$ kPa

**Fig. 11:**
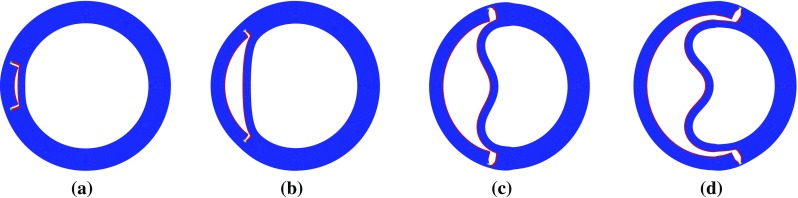
Steady deformed configurations with $\alpha =160^\circ$ and different lengths $\eta$ after dissection propagation: **a** $\eta =30^\circ$, **b** $\eta =90^\circ$, **c** $\eta =150^\circ$, and **d** $\eta =210^\circ$. All the tears propagate radially

Our previous study on a 2D strip (Wang et al. 2015) showed that tear length plays an important role in the dissection; a longer tear is always more likely to propagate. We now show that this is no longer true for a circular geometry and when residual stress is present. Three additional groups of simulations were carried out with $\eta =30^\circ ,~150^\circ$, and $210^\circ$, and some interesting results are found when tear length increases. The deformed configurations for $\alpha =160^\circ$, Fig. 10, show that buckling of the inner wall (the material section between the lumen and tear) occurs for $\eta = 150^\circ$ and $\eta =210^\circ$. The maximum principal stress distribution and the critical pressure are also shown in Fig. 10. Beyond the cohesive criterion, the tear propagates. All tear propagations tend to be radially outwards, as shown in Fig. 11.

**Fig. 12:**
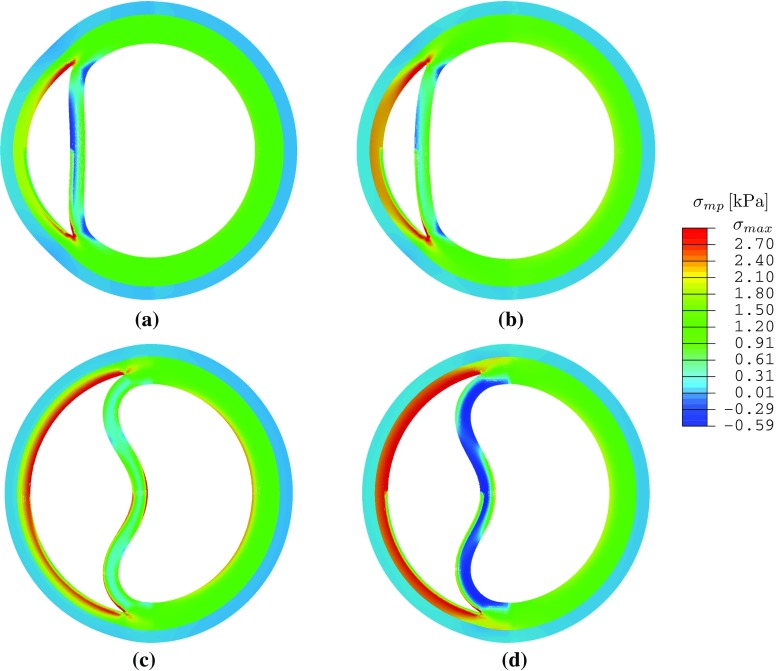
The maximum principal stress in the deformed configuration at the critical pressure $p'_\mathrm{c}$ is plotted for $\eta =90^\circ$ (*above*) and $\eta =150^\circ$ (*below*), with $(\alpha =40^\circ )$ and without $(\alpha =0^\circ )$ residual stress. **a** $\eta = 90^\circ$, $\alpha = 0^\circ$, $p^\prime _c = 0.22$, $\alpha _\mathrm{min} = -0.37$ kPa, $\sigma _\mathrm{max} = 3.35$ kPa. **b** $\eta = 90^\circ$, $\alpha = 40^\circ$, $p^\prime _c = 0.27$ $\sigma _\mathrm{min} = -0.28$ kPa, $\sigma _\mathrm{max} = 3.35$ kPa. **c** $\eta = 150^\circ$, $\alpha = 0^\circ$, $p^\prime _c = 0.23$ $\sigma _\mathrm{min} = -0.58$ kPa, $\sigma _\mathrm{max} = 4.88$ kPa. **d** $\eta = 150^\circ$, $\alpha = 40^\circ$, $p^\prime _c = 0.25$ $\sigma _\mathrm{min} = -0.59$ kPa, $\sigma _\mathrm{max} = 4.90$ kPa

Comparison of the critical pressure for different tear lengths (Fig. 9) shows that the dimensionless critical pressure $p'_\mathrm{c}$ increases with $\alpha$ in all the cases simulated. Notably, the longest tear length studied $(\eta =210^\circ )$ has a higher value of $p'_\mathrm{c}$ than that of $\eta =90^\circ$, and $150^\circ$. Hence, the relationship between propagation and tear length is not as simple as that of a 2D strip (Wang et al. 2015). This presumably is due to the buckling of the inner wall, which is more likely to occur for a longer tear. However, for the intermediate case, $\eta =90^\circ$, inner wall buckling occurs only for $\alpha =0^\circ$, and not when $\alpha \ge 40^\circ$ (Fig. 12). This suggests that there is a subtle interplay between dissection length, residual stress, and inner wall buckling.

### The effect of fibre orientation

Here we vary the fibre orientation. We refer to the fibre orientation listed in Table 2 as the physiological or ‘true’ case. In the following simulations, we assume that the media and the adventitia have the same fibre orientation, so that $\beta _\mathrm{m}=\beta _\mathrm{a}=\beta$. Simulations were performed with $\beta =0^\circ ,~10^\circ ,~15^\circ ,~20^\circ ,~30^\circ ,~60^\circ$ and $90^\circ$. In addition, a group of simulations were run without fibres $(k_1=0)$, referred to as the ‘free’ case.

**Fig. 13:**
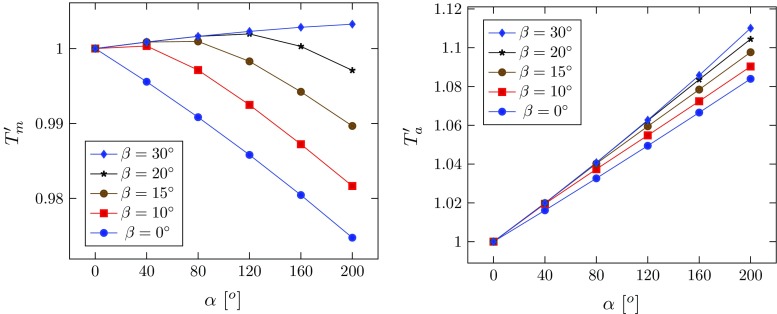
Wall thickness $T_\mathrm{m}'$ and $T_\mathrm{a}'$ for the media and adventitia in $\varOmega _0$ normalized with respect to their values in $\varOmega _\mathrm{r}$, plotted against the opening angle for different fibre angles. For other fibre angles ($\beta =~60^\circ ,~90^\circ$, free, true), the curves overlap that for $\beta =30^\circ$

To ensure that the differences between the simulations are only due to the fibre orientations, we use the same unloaded configuration $\varOmega _r$, with $\varOmega _0$ calculated for each fibre orientation using the analytical method. The normalized thicknesses of the media and adventitia, $T_\mathrm{m}'=T_\mathrm{m}/ t_\mathrm{m}$ and $T_\mathrm{a}'=T_\mathrm{a}/t_{\mathrm{a}}$, are plotted against the opening angle $\alpha$ in Fig. 13, for the different fibre orientations. The thickness of the media $T_\mathrm{m}'$ decreases with the opening angle for smaller values of $\beta$ (e.g. when $\beta =0^\circ$, or $10^\circ$), but the trend changes as $\beta$ increases. When $\beta \ge 30^\circ$, it increases monotonically. On the other hand, the thickness of the adventitia $T_\mathrm{a}'$ increases monotonically with the opening angle. For other fibre angles ($\beta =~60^\circ ,~90^\circ$, free, true), the results are identical to that of $\beta =30^\circ$. This is because fibres beyond this angle are no longer stretched, i.e. $I_4<1$.

**Fig. 14:**
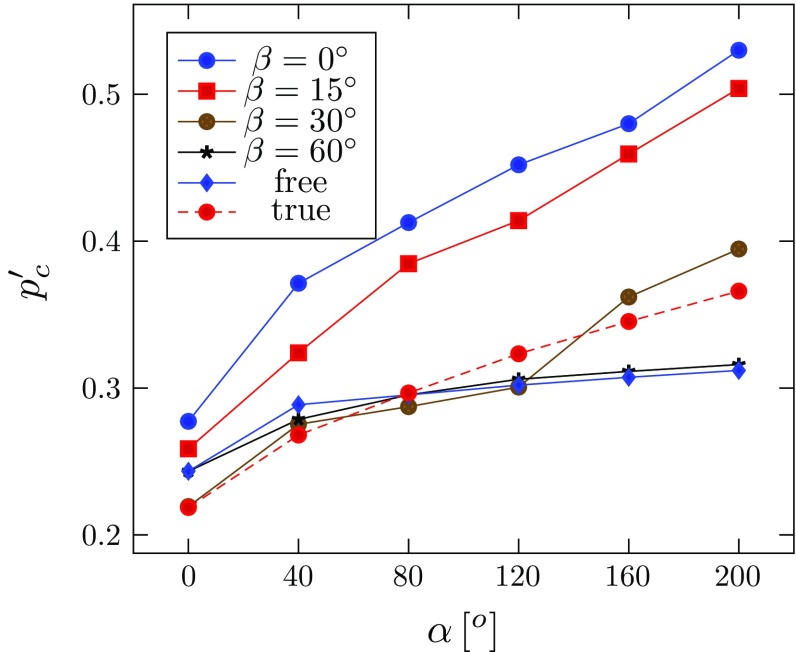
The dimensionless critical pressure, $p'_\mathrm{c}$, is plotted against the opening angle, $\alpha$, for different fibre orientations. The results for $\beta \ge 60^\circ$ are identical to that of $\beta =60^\circ$, and the ‘free’ case, indicating that the fibres at these angles do not bear load

The critical pressure also changes with different fibre orientation (Fig. 14). Notice that with the inflation, fibres with $\beta =30^\circ$ also start to bear load, but fibres at greater angles $(\beta =60^\circ$ and $90^\circ )$ still do not take on any load, and hence, the critical pressures for these cases remain the same as in the ‘free’ case. As expected, the critical pressure when $\beta =0^\circ$ is the highest, since the residual stress is the greatest in this case.

## Discussion and conclusions

**Fig. 15:**
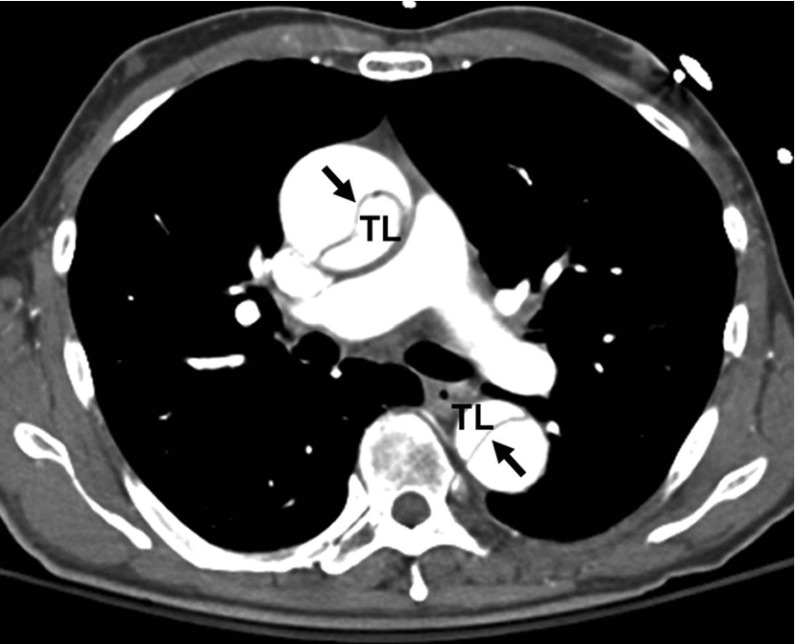
This CT shows an acute aortic dissection in the ascending and descending aorta (Fig. 2; Braverman 2010), with buckling of the inner wall indicated by the *arrows*. TL is the true lumen

We have used both analytical and computational approaches to study the effect of residual stress on the propagation of arterial dissection. Our simulations show that the shortest $(30^\circ )$ and the longest tear $(210^\circ )$ are the most stable (with higher critical pressures), while tears of lengths $150^\circ$ and $90^\circ$ are most unstable. However, residual stress increases the critical pressure in all cases; the most dramatic improvement is seen for the $90^\circ$ tear. This suggests that both the length of tear and the residual stress play an important role in determining the critical pressure for the tear propagation. In particular, we found interesting inner wall buckling associated with the longer tears. Similar buckling was observed in a computed tomography (CT) scans of a patient in the ascending aorta (Fig. 15). Clearly, there is an intricate balance between tear length, buckling of the inner wall, fibre orientation, and residual stress, all of which may affect the likelihood of tear propagation. One plausible explanation is that, although a longer tear propagates more readily, it is also more likely to cause the inner wall to buckle and so increase the value of $p_\mathrm{c}$. However, the exact mechanism remains to be explored.

**Fig. 16:**
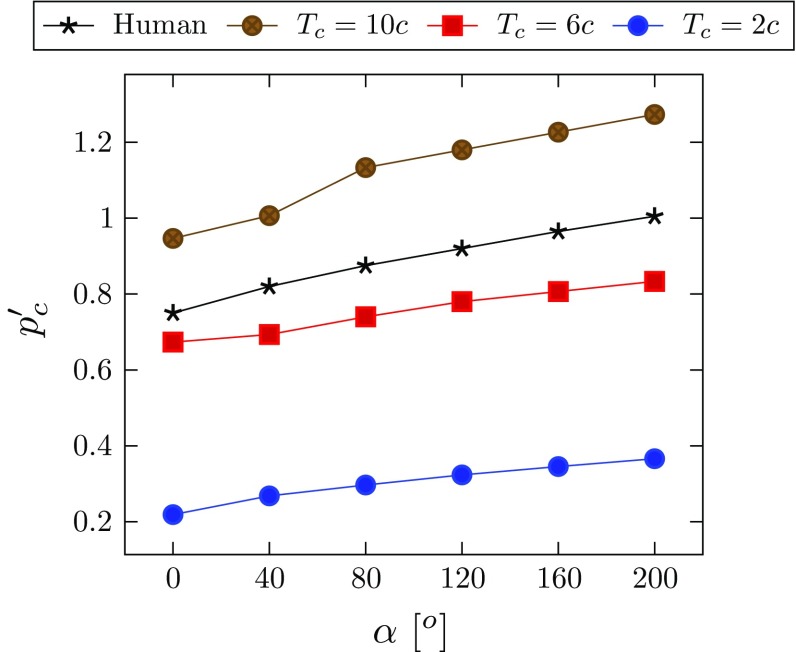
The dimensionless critical pressures $p'_\mathrm{c}$ against the opening angle $\alpha$, for the rabbit carotid artery at different values of $T_\mathrm{c}$, as well as for the aged human thoracic aorta $(T_\mathrm{c}=2c.)$

**Table 5:** The model parameters used for the human thoracic aorta in $\varOmega _0$ (Fereidoonnezhad et al. 2016)

	$c(\hbox {kPa})$	$k_1\,(\hbox {kPa})$	$k_2$	$\beta \,(^\circ )$	$T_\mathrm{c}$	$G_\mathrm{c}(\hbox {N/m}^2)$	$\alpha \,(^\circ )$	$T_\mathrm{i}(\hbox {mm})$	$R_\text {i}(\hbox {mm})$
Media	20	112	20.61	41	2	0.001	80	0.69	1.13
Adventitia	8	362	7.089	50.1	2	0.0001	80	0.48	N/A

We now discuss the limitations of this study. Our model is a plane-strain problem and does not include the effect of axial stretch. In our previous work (Wang et al. 2015), we found that axial stretching of fibres resists the opening of the dissection and significantly decreases the energy release rate for tear propagation. Clinically, dissections may propagate axially and may re-enter the lumen. Our model also cannot predict the absolute value of the critical pressure due to the simplifications mentioned above and lack of data on cohesive parameters, e.g. the value of $T_\mathrm{c}$. Nevertheless, this model provides a qualitative description of the variation of the critical pressure with the residual stress. This is further illustrated in Fig. 16 for $T_\mathrm{c}/c=2$, 6, 10 for the rabbit carotid artery, as well for the aged human thoracic aorta, where the shear modulus of the media is much greater, as shown in Table 5. In the human artery model, all the parameters for the HGO model (Table 5) are estimated by fitting the experimental data of the cyclic uniaxial tensile tests of 14 human thoracic aortas ($60\pm 12$ year, $\hbox {mean}\pm \hbox {SD}$) (Fereidoonnezhad et al. 2016). Our results show that changes of the critical pressure with residual stress are similar for human aorta and rabbit carotid artery.

The other limitation is that we use an isotropic cohesive traction–separation law. The tensile testing of a porcine thoracic aorta performed by MacLean et al. (1999) showed that the stiffness in the radial direction is significantly lower than in the circumferential and longitudinal directions. MacLean et al. (1999) also performed a histological analysis to show the behaviour of elastin layers and smooth muscle cells during the aortic dissection. Work is ongoing to develop a three-dimensional and anisotropic arterial dissection model that could make use of the histological data.
